# Therapeutic challenge in delusional disorder: a case report and literature review

**DOI:** 10.1192/j.eurpsy.2022.1979

**Published:** 2022-09-01

**Authors:** P. Trindade, C. Laginhas, C. Adão, H. Canas-Simião, A. Ribeirinho Marques, R. Caetano

**Affiliations:** Centro Hospitalar de Lisboa Ocidental, Psychiatry Department, Lisboa, Portugal

**Keywords:** Treatment, Psychosis, Delusional disorder, antipsychotic

## Abstract

**Introduction:**

Delusional disorder (DD) is defined by the presence of one or more delusions, of at least one month’s duration, in the absence of prominent hallucinations or other symptoms of schizophrenia. Although functioning may not be markedly impaired, the delusion(s) or its ramifications may have a significant impact in the patient’s life. With a life-time prevalence of 0.18%, DD is still neglected in terms of approved treatment recommendations.

**Objectives:**

We present the case of a patient diagnosed with DD and discuss the treatment of DD according to current evidence.

**Methods:**

Relevant clinical information was extracted from the patient’s clinical process. A non-systematic review was made in Pubmed database with the terms “Delusional Disorder” and “Treatment”.

**Results:**

Male, 76 years old, divorced, living alone, autonomous. First admitted at age 62 in our inpatient psychiatry ward for a persecutory delusion regarding his neighbors. He was discharged with the diagnosis of DD and started a follow-up in a mental health community team. He abandoned treatment and psychiatric consultation after 9 years. During 17 years he moved home more than 10 times due to a progressive dynamism of the delusion, leading to recent marked behavior changes towards his neighbors. He is again admitted in our inpatient psychiatry ward.

**Conclusions:**

This case illustrates the impact that untreated DD can have on its patients. Although consensus using antipsychotics, there are still insufficient studies to make evidence-based recommendations to treat people with DD. Further research is needed in this sense.

**Disclosure:**

No significant relationships.

